# Case report: Use of therapeutic drug monitoring and pharmacogenetic testing as opportunities to individualize care in a case of flecainide toxicity after fetal supraventricular tachycardia

**DOI:** 10.3389/fped.2023.1168619

**Published:** 2023-06-28

**Authors:** Ronald Palmen, Tracy Sandritter, Lindsey Malloy-Walton, Christopher Follansbee, Jonathan B. Wagner

**Affiliations:** ^1^ Children’s Mercy, Kansas City, MO, United States; ^2^ Department of Pediatrics, University of Missouri-Kansas City School of Medicine, Kansas City, MO, United States; ^3^ University of Missouri-Kansas City School of Medicine, Kansas City, MO, United States; ^4^ University of Missouri-Kansas City School of Pharmacy, Kansas City, MO, United States; ^5^ Ward Family Heart Center, Kansas City, MO, United States; ^6^ Division of Clinical Pharmacology, Toxicology and Therapeutic Innovation, Kansas City, MO, United States

**Keywords:** flecainide, pharmacogenetic, pharmacogenomics, toxicity, drug monitoring

## Abstract

Flecainide is a class IC antiarrhythmic utilized in prophylaxis of refractory paroxysmal supraventricular tachycardias in pediatric populations. Despite being a highly effective agent, its narrow therapeutic index increases the risk of toxicity and proarrhythmic events, including wide-complex tachycardia. In the absence of direct plasma sampling in the fetus to quantitate flecainide systemic concentrations, clinicians typically make drug dosing decisions from maternal plasma concentrations and QRS duration on maternal ECGs. There remains a paucity of standard guidelines and data to inform the timing and frequency of the aforementioned test in pregnancy and timing of flecainide discontinuation prior to childbirth. Flecainide primarily undergoes metabolism via cytochrome P450 (CYP). Given the variance of CYP-mediated metabolism at the level of the individual patient, pharmacogenomics can be considered in patients who present with flecainide toxicity to determine the maternal vs. fetal factors as an etiology for the event. Finally, pharmacogenetic testing can be utilized as an adjunct to guide flecainide dosing decisions, but must be done with caution in neonates <2 weeks of age. This case report highlights utilization of pharmacogenomic testing and therapeutic drug monitoring as adjuncts to guide therapy for a newborn with refractory supraventricular tachycardia, who experienced flecainide toxicity immediately post-partum and was trialed unsuccessfully on multiple alternative antiarrhythmics without rhythm control.

## Introduction

Flecainide acetate is a class IC antiarrhythmic that works primarily by blocking Na+ channels and therefore used for paroxysmal supraventricular tachycardias (SVT), including atrioventricular (AV) nodal re-entrant tachycardia, AV re-entrant tachycardia, atrial tachycardia, and atrial fibrillation/flutter in patients who do not have structural heart disease. However, flecainide has a narrow therapeutic index with resultant wide-complex tachycardia and the possibility of death if toxicity is not recognized and effectively treated. Fortunately, neonatal flecainide toxicity is rare, and even more so, toxicity immediately post-partum is virtually undocumented. Toxicity in the neonatal period may be secondary to a multitude of factors including maternal, maternal–fetal, or neonatal factors. In terms of maternal pharmacokinetic factors, such as absorption, distribution, metabolism, and excretion, there is an absence of data to inform recommendations on timing of the discontinuation of flecainide prior to delivery. Additionally, neonatal factors must all be addressed in the event of suspected toxicity as evidenced by this case, including variance in drug metabolism. Flecainide is primarily metabolized by CYP2D6, which is known to be highly polymorphic, and could impact flecainide exposure in the neonatal patient. Our case presents the utilization of pharmacogenomic testing and therapeutic drug monitoring in a patient with flecainide toxicity immediately post-partum as a means to thoughtfully reintroduce the agent given his complex, medication-resistant SVT. Pharmacogenetic testing is an option in the precision pharmacotherapy toolset that needs further elucidation in the pediatric population, but shows promise as a means to individualize drug dosing decisions.

## Case report

The patient is a Caucasian male born at 37 weeks 1 day gestation with history of fetal tachycardia to a previously healthy 28-year-old G1P0 female. Pregnancy was complicated by fetal tachycardia (suspected SVT) detected at 32 weeks and 1 day during a routine obstetric appointment. Her pregnancy time course is summarized in Table 1. Initially, she was given oral digoxin, followed by maintenance dosing at an outside facility. She was also started on antenatal corticosteroids in preparation of possible early delivery. Two days following digoxin initiation, the fetus remained in predominant tachycardia and frequent premature atrial contractions with brief episodes of likely sinus rhythm. Therefore, the mother was transferred to our facility and referred to fetal cardiology for further management. The fetal echocardiogram revealed a structurally normal heart with normal biventricular size and absence of fetal hydrops. The rhythm had 1:1 AV conduction and a rate of 180–200 beats per minute (bpm) with a likely long RP morphology. Fetal heart rate monitoring displayed heart rates in the 190 bpm for >50% of the total beats. The mother's baseline electrocardiogram displayed sinus bradycardia with a QRS duration of 88 ms, and baseline maternal echocardiography revealed no structural abnormalities and a normal left ventricular ejection fraction (∼55%–60%). Her serum digoxin trough level was 0.88 ng/ml (reference range: 1–2 ng/ml). Given the failure of response to digoxin, the mother was transitioned to flecainide 100 mg three times a day until delivery. A plasma flecainide trough concentration was obtained after the fifth dose that was within normal limits at 0.23 µg/ml (reference range: 0.2–1.0 µg/ml). Importantly, there was only an 11% increase in the maternal QRS duration on daily electrocardiograms over 5 days while hospitalized. Four days following flecainide initiation, the fetus displayed tachycardia for <50% of the total beats and was in a sinus rhythm on repeat fetal echocardiography. Given the response to treatment, the mother was discharged home with close follow-up.

**Table 1:** Diagnostic and therapeutic time course during pregnancy.

Age	Clinical finding	Maternal QRS duration	Flecainide serum concentration	Intervention
32 wks 1 d gestation	Fetal SVT diagnosed			Digoxin load and maintenance + corticosteroids
32 wks 3 d gestation	Predominant tachycardia and frequent PACs with brief episodes of likely sinus rhythm			Referred to our facility
32 wks 5 d gestation	Maternal ECHO normal with LVEF (∼55%–60%) Fetal long RP tachycardia (∼180–200 bpm) with 1:1 AV conduction	88 ms		Flecainide 100 mg PO TID
32 wks 6 d gestation		97 ms	0.23 µg/ml (range:0.2–1.0 µg/ml)	
33 wks 2 d gestation	fetus displayed tachycardia for <50% of the total beats and was in a sinus			Mother was discharged home with close follow-up.
36 wks 6 d gestation	Routine screening maternal ECG	124 ms (increase 40% from baseline)	None obtained	

wks, weeks; d, days; SVT, supraventricular tachycardia; PACs, premature atrial contractions; ECHO, echocardiogram; LVEF, left ventricular ejection fraction; AV, atrial ventricular; PO, by mouth; TID, three times daily dosing; ECG, electrocardiogram.

**Figure 1:**
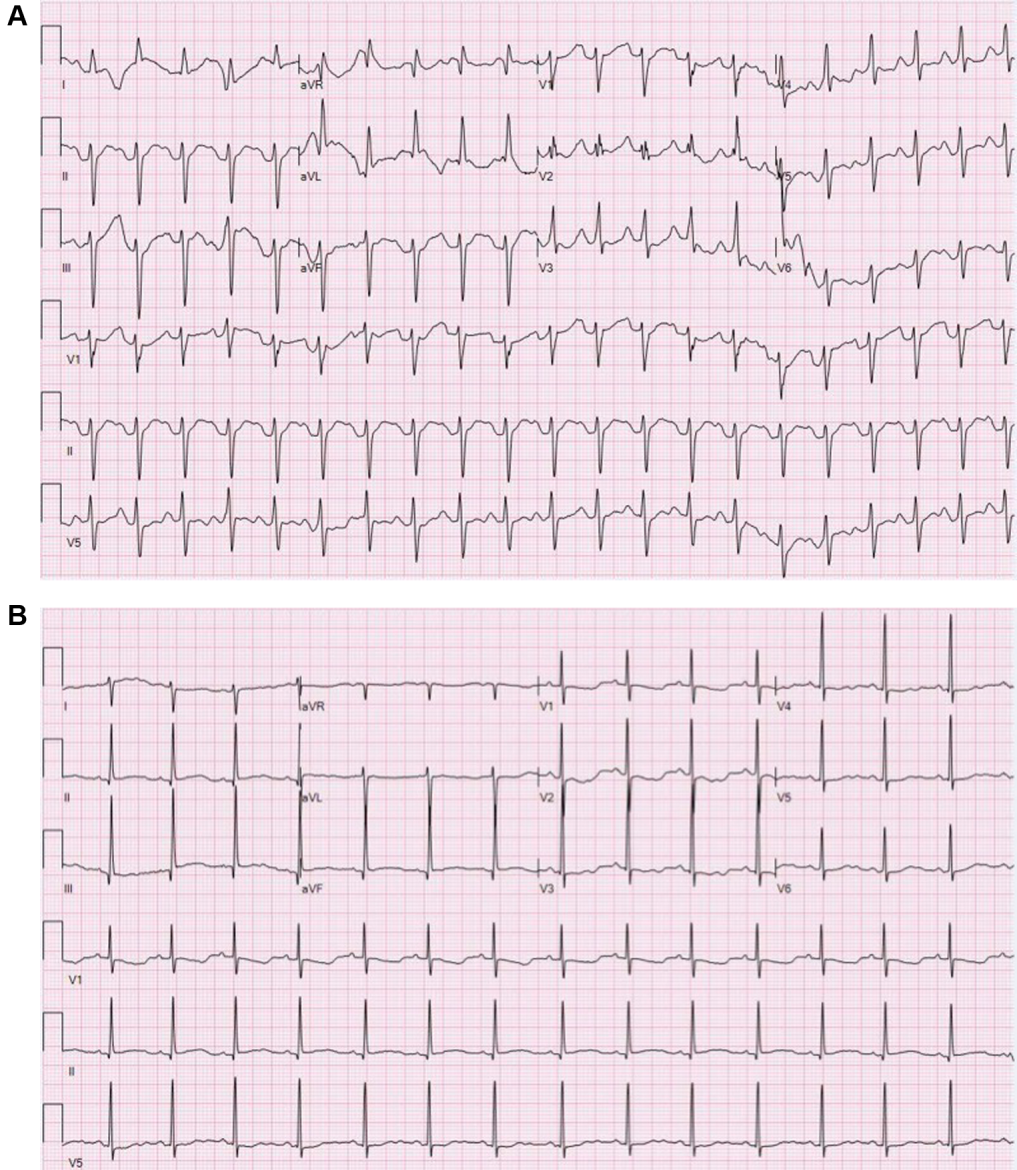
(**A**) Electrocardiogram during flecainide toxicity demonstrating 1:1 AV conduction with a prolonged PR interval, left axis deviation, nonspecific intra-ventricular conduction block and prolonged QTC. Rate 125 bpm, PR interval 190 ms, QRS duration 100 ms, QTc 494 ms. (**B**) Electrocardiogram following sodium bicarbonate treatment demonstrating sinus bradycardia with normalization of the QRS duration, diffusely flat *T* wave changes and prolonged QTc. Rate 88 bpm, PR interval 146 ms, QRS duration 70 ms, QTc 529 ms.

**Figure 2:**
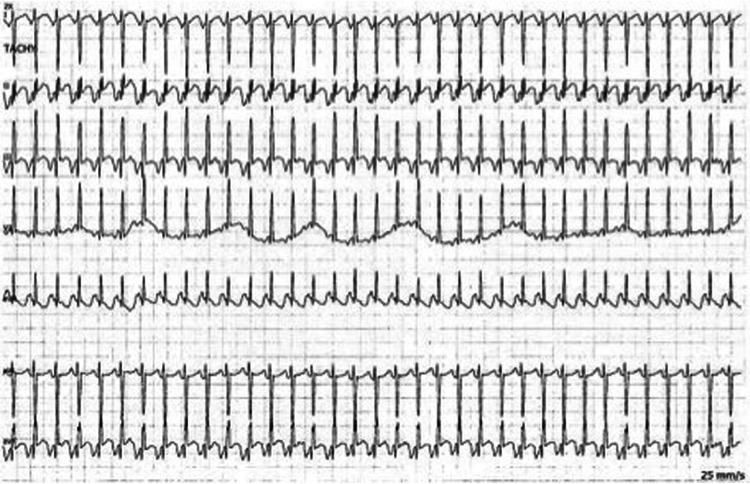
Bedside telemetry demonstrating a regular, narrow complex tachycardia with long RP morphology and deeply negative *P* waves in the inferior leads with PJRT.

At 37 weeks 1 day gestation, the mother presented to our facility for planned induction. She had an electrocardiogram 3 days prior with a QRS duration of 124 ms (∼40% increase from baseline). No maternal plasma flecainide trough concentration was obtained at this time. She took her last dose of flecainide at 0600 the morning of admission. The infant was delivered roughly 13 h later. At birth, the gross and histological examination of the placenta did not reveal any abnormalities or degenerative changes. After delivery, he was noted to have a poor respiratory effort and grunting. His APGAR scoring was 1, 5, and 8 at 1, 5, and 10 min, respectively. His birth weight was 3 kg and that value was used for his calculation weight throughout his hospitalization. Cardiac telemetry initially identified an irregular predominantly wide-complex rhythm with long RP morphology, occasional narrow complex beats, and frequent wide-complex couplets and triplets at a rate of 150 bpm (Figure 1A). An echocardiogram revealed preserved ejection fraction of 59%. For diagnostic purposes, a dose of adenosine (0.2 mg/kg) was given by rapid push followed by 10 ml normal saline that resulted in a momentary change from wide to narrow QRS morphology with slowing of the rate suggesting sinus rhythm with aberrant AV conduction. Flecainide toxicity could not be excluded in the presence of rate-dependent aberrancy during sinus rhythm, and therefore, 1 mEq/kg of sodium bicarbonate was administered leading to normalization of the QRS duration (Figure 1B). A serum flecainide plasma level was not obtained during this acute event. He was then monitored off any antiarrhythmic therapy until the third day of life when the infant had recurrence of paroxysmal tachycardia. Several episodes began with aberrantly conducted wide-complex beats, consistent with the Ashman phenomenon, followed by narrow complex tachycardia of long RP morphology with deeply negative *P* waves in the inferior leads and rates 180–220 bpm consistent with a rare and often medically refractory form of SVT called persistent junctional reciprocating tachycardia (PJRT) (Figure 2). The infant was started on oral propranolol 0.75 mg every 6 h (1 mg/kg/day) and titrated to 1.5 mg every 6 h (2 mg/kg/day) over the course of a 12 h period. At this time, the majority of the episodes of breakthrough narrow complex tachycardia were less than 30 s in duration and self-resolving. By day 5, there was increased frequency of narrow complex tachycardia warranting a 500 μg/kg esmolol bolus over 1 min and a continuous esmolol infusion of 100 μg/kg/min. Despite titration to 300 μg/kg/min over the course of 28 h, the infant continued to have breakthrough tachycardia and was transitioned to intravenous sotalol 3 mg every 8 h (3 mg/kg/day) infused over 1 h. This dose was increased on day of life 7 to 5 mg every 8 h (5 mg/kg/day) infused over 5 h due to continued breakthrough tachycardia.

Of note, on day 7 of life, there was blood noted in the diaper and the patient's oral feeding was stopped for concerns of necrotizing enterocolitis. However, the screening x-ray, complete blood count with differential, C-reactive protein, and the physical examination were within normal limits. He was given bowel rest for 48 h and no antimicrobials were given. Sotalol was discontinued on day 9 of life due to continued breakthrough SVT and he was transitioned to amiodarone (2.5 mg/kg bolus, then 10 mg/kg/day infusion). Given the persistence of the breakthrough episodes of SVT on multiple medications and the previous treatment success with flecainide during gestation, pharmacogenomic testing was performed given the history of flecainide toxicity and concerns for altered CYP2D6 metabolism (RightMed®, OneOme, Minneapolis, MN, United States). For the next several days, he continued to experience breakthrough SVT and required additional amiodarone boluses. By the 12th day of life, amiodarone infusion was increased to 20 mg/kg/day and esmolol was restarted at 100 μg/kg/min and titrated up to 400 μg/kg/min over 34 h. Despite these efforts, he continued to have breakthrough episodes of SVT. Given a concern for prior toxicity, flecainide was initiated at the lower end of normal dosing range, 3 mg every 8 h (3 mg/kg/day). Close monitoring with reintroduction of flecainide was observed with continuous telemetry and daily electrocardiograms, which did not reveal QRS widening. A flecainide trough concentration was obtained 8 h after the sixth dose and was 0.2 µg/ml (reference range: 0.2–1.0 µg/ml). With the reported elimination half-life of flecainide being 29 h immediately after birth and 11–12 h in children under 1 year of age (1), this would represent a range of ∼1.5 to ∼4.5 half-lives, respectively. At 15 days of life, flecainide was increased to 4 mg every 8 h (4 mg/kg/day) given breakthrough tachycardia. Over the next 5 days, he had improved control of his tachyarrhythmia and his QRS duration remained stable ranging from 70 to 76 ms. Therefore, amiodarone was discontinued and oral sotalol was initiated again at 5 mg every 8 h (5 mg/kg/day). Two days following sotalol initiation, he was weaned off esmolol. His baseline QTc at time of sotalol initiation was 476 ms and remained stable throughout the remainder of his hospital course (range: 430–475 ms). His pharmacogenomic testing returned at 22 days of life, which revealed a CYP2D6 *1/*3 genotype, consistent with intermediate metabolism phenotype (active score = 1). Additionally, the patient had CYP1A2 *1A/*1F genotype, consistent with rapid metabolism phenotype, which is the most commonly observed variation. In the absence of available data to inform pharmacogenetic-driven guidelines for dosing in the pediatric population, our precision medicine consult service (GOLDILOKs©) recommended guidance consistent with the Dutch Working Group Guidelines for CYP2D6 intermediate metabolizers, utilizing 75% of the standard dose. With the higher end of flecainide dosing being 6 mg/kg/day, flecainide dosing was increased to 4.5 mg every 8 h (4.5 mg/kg/day) consistent with a 75% reduction. A repeat flecainide concentration 7 h after the fifth dose remained low at 0.1 µg/ml. The QRS duration also remained stable with the adjustment (range: 62–70 ms). The infant was able to be discharged at 26 days of life on flecainide (4.5 mg/kg/day) and sotalol (5 mg/kg/day). At 12 and 14 months, the patient was successfully weaned off sotalol and flecainide, respectively, without any episodes concerning for breakthrough tachycardia. To date, the patient has not required hospitalization since discharge.

## Discussion

We describe a case of a term infant with history of fetal SVT that presented with suspected peripartum flecainide toxicity stabilized with sodium bicarbonate and successfully re-initiated on flecainide in conjunction with sotalol for longer-term arrhythmia management of refractory paroxysmal junctional reciprocating tachycardia. Utilization of flecainide in fetal supraventricular tachycardia has more recently been described as a treatment strategy either as a dual therapy or monotherapy (2–6). Although the algorithms may be center-specific, but generally dosing at 300/day is recommended. Flecainide toxicity is a rare occurrence in the neonatal population that has been associated with dosing errors (7, 8), at times of dietary changes (9), suspected impaired metabolism (10), or without clear etiology (11, 12). Although reports of peripartum flecainide toxicity have been described (13), no cases have reported the use of therapeutic drug monitoring and pharmacogenetic testing as guides for reinitiating flecainide therapy for rhythm control. Only one small cohort has simultaneously compared maternal and fetal flecainide plasma concentrations and that study indicates ∼80% transplacental transfer (6). However, the range of fetal concentrations was large (e.g., 400–800 µg/L) (6).

Our case highlights important points with regard to the management of fetal SVT and flecainide utilization. The etiology of flecainide toxicity remains ambiguous in this case but likely related to intrinsic maternal-specific factors, maternal–fetal factors later in pregnancy, or patient-specific factors. Herein, we will describe each of these possible contributing factors, supplemented with what exists in the literature.

**Maternal factors** could have led to increased flecainide systemic exposure and toxicity in the infant. In particular, the maternal QRS duration on ECG had a 40% increase compared to baseline prior to delivery. Unfortunately, there was not a corresponding flecainide plasma concentration obtained with this change on the electrocardiogram just prior to delivery. Given the increase in QRS duration, it prompts the question whether the maternal plasma concentration was higher at this time in the pregnancy. From a drug disposition perspective, available evidence in humans suggests that clearance by CYP2D6 is increased in pregnancy (14), a situation where decreased systemic exposure to flecainide would potentially occur. Conversely, CYP1A2, a drug metabolism enzyme contributing to a smaller degree of flecainide clearance (15), activity is reduced during pregnancy (16, 17). It is conceivable that the mother is an intermediate or poor metabolizer of CYP2D6, and despite enhanced renal and hepatic clearance that typically occur during pregnancy, the genotype–phenotype relationship would lead to increased systemic exposure. Of note, this mother did not have other symptoms suggestive of flecainide toxicity. Although with the aforementioned electrocardiographic changes, it cannot be excluded that her flecainide systemic exposure was increased. Given there exists a high correlation between maternal and fetal plasma concentrations (18), this could have contributed to a higher flecainide systemic exposure in the fetus. Therefore, we would strongly recommend therapeutic drug monitoring, in conjunction with serial electrocardiogram monitoring, especially nearing parturition in cases of fetal SVT treated with flecainide therapy. One could argue that *CYP2D6* genotype status would additionally be valuable in those patients where plasma concentrations are elevated. Specifically with the case presented, guided drug level monitoring in late pregnancy would hold the potential to expedite recognition and understanding of drug levels outside of therapeutic range and assist guiding further management of fetal tachyarrhythmias following delivery through early hours and days. This would virtually eliminate the delay adequate management and SVT burden.

Alternatively, **maternal–fetal factors** and, in particular, changes in maternal–fetal physiology (e.g., transplacental distribution/transfer) could be contributing factors to the case. One of the major points of discussion this case highlights is the optimal management of antiarrhythmic, namely, flecainide, dosing in peripartum management of SVT. There exists a paucity of supporting evidence or consensus guidelines regarding optimal dosing of flecainide and timing of discontinuation of these agents prior to delivery and, therefore, guidelines are typically provider- or center-specific (2–6). This is in part due to the scarcity of cases, in which flecainide is the antiarrhythmic of choice, to properly inform a prospective randomized trial regarding timing of discontinuation. Other antiarrhythmics previously utilized for fetal tachyarrhythmias, including amiodarone albeit having a much longer half-life (estimated 30 days) (19) compared to flecainide (12–27 h) (20, 21) are recommended to taper weeks to days prior to delivery (22). A similar argument could be plausibly made for flecainide where discontinuation 3–4 half-lives prior to delivery (∼36–48 h in the pregnancy state of enhanced clearance) should be made to avoid concerns of toxicity at birth. It has been observed that maternal and fetal plasma flecainide concentrations correlate well in the third trimester of pregnancy (18). Post-partum, the neonate could be prone to a decrease in flecainide clearance with separation from enhanced maternal renal and hepatic clearance pathways, leading to increased systemic exposure and a toxic state, which could have been a factor in our case. This evidence suggests that our aforementioned recommendation of therapeutic drug monitoring through pregnancy until delivery is warranted when using flecainide therapy. Additionally, maternal pharmacogenetic testing to determine how elimination half-life will be affected who allow the care team opportunities to determine fetal exposure to flecainide and create plans for tapering, discontinuation, and/or dose alterations during pregnancy.

Finally, **patient-specific factors** in the neonate could have led to this case of flecainide toxicity. Diminished flecainide clearance via CYP2D6-mediated biotransformation in the neonate could have led to an increase in systemic exposure and, therefore, risk of flecainide toxicity. CYP2D6 contributes to approximately 25%–30% of drugs used clinically and is one of the most polymorphic drug metabolizing enzymes (23–25). Therefore, systemic exposure of CYP2D6 drug substrates in the setting of genotypes associated with poor metabolism could lead to increased systemic exposure (26). Pharmacogenetics is an emerging field that focuses on patient-specific factors (e.g., drug metabolism enzyme genotypes) to optimize medication dosing (27). As pharmacogenetics and pharmacogenomics progress toward implementation to the clinical space, one can anticipate the desire for pharmacogenetic-driven guidelines of medication management tailored to each individual pediatric patient would be of value (28, 29), similar to guidance delivered to adult cohorts from the Dutch Pharmacogenetics Working Group (DPWG) and the Clinical Pharmacogenetics Implementation Consortium (CPIC) (30, 31). However, this genotype–phenotype extrapolation to the growing child may be challenging, especially given the distinct ontogenic changes in expression of CYP enzymes in the developing child (32, 33). Therefore, the genotype–phenotype relationship may not be applicable to this case involving a neonate where CYP2D6 was still immature. In fact, available *in vivo* data suggest that CYP2D6 expression may not mature until at least 2 weeks of age (34–36). For neonates with immature CYP2D6 expression, could a genotype consistent with intermediate metabolism functionally behave similar to older patients with poor metabolism? Collectively, there remains a dearth of data related to the impact of CYP2D6 genotype on flecainide exposure (i.e., pharmacokinetics) and thereby response (i.e., pharmacodynamics), but needs to be prospectively evaluated. In our case we do know that the pharmacogenetic testing that resulted over 2 weeks of age may have not directly contributed to the etiology of flecainide toxicity (i.e., no genotype–phenotype relationship was observed), but helped guide and give reassurance to our electrophysiologist on appropriate dosing of flecainide after a history of flecainide toxicity. Finally, further understanding is necessary in the role of CYP1A2 in flecainide metabolism as well. *In vitro*, CYP1A2 contributes to flecainide metabolism to an m-O-dealkylated flecainide (MODF), but only one-sixth of that compared to CYP2D6 (15). Additionally, CYP1A2 has very low expression in infancy (33), likely making the patient's genotype of rapid metabolism irrelevant. However, prospective data to determine its contribution in the face of diminished CYP2D6 expression as noted in this case should be evaluated. In the future, one could also explore gene mutations associated with drug targets as etiologies for flecainide toxicity, namely, *SCN5A* where selected gene variants have predicted QRS prolongation when challenged with sodium channel blocker (e.g., flecainide) (37, 38). The commercial pharmacogenomic platform used in this case report does not have *SCN* gene mutations included, but should be consider in future expansions of the panel. Ultimately, physiologically based pharmacokinetic modeling could provide any opportunity to mechanistically evaluate flecainide exposure in the setting of variable CYP2D6/CYP1A2 expression due to genetic variation and/or ontogeny and inform clinical trial design in the neonate requiring flecainide therapy (39). Pharmacogenetic data did not provide the etiology of flecainide toxicity, but can serve as an adjunct tool in the management of complex pediatric and maternal patients dosed with CYP2D6 drug substrates.

## Lessons learned

The case presented exemplifies the growing possibilities of pharmacogenetic testing in the clinical realm while exposing the need for further understanding of the intricacies of early expression of drug disposition pathways. Although testing can be performed in infants less than 2 weeks of age, the result may be unreliable until they are older than 2 weeks of age. As with this case, we suggest considering therapeutic drug monitoring in patients taking flecainide, yet it is critical to perform testing once under steady state, which varies based off of neonate age. It is estimated to be closer to 80 h following drug administration in a neonate less than 1 month of age, assuming T_1/2_ ∼20 h and steady state occurs following four half-lives, which declines closer to 48 h with advanced clearance and half-life of 12 h following 1 month of age. In theory, this is a result of maturation of CYP2D6 and its ability to clear flecainide more efficiently. It could prove beneficial to investigate the early maturation of these enzymes with prospective trials with pharmacogenetic status to inform use and guidelines on drug administration in the future.

## Disclosures

The authors declare no conflicts of financial interest in any product or service mentioned in the manuscript, including grants, equipment, medications, employment, gifts. The authors had full access to all patient information in this report and take responsibility for the integrity and accuracy of the report. In accordance with our institutional guidance, Authorization for Presentation of Case Report was obtained prior to development of this manuscript.

## Data Availability

The original contributions presented in the study are included in the article, further inquiries can be directed to the corresponding author.
